# Biomarkers in patients with clinical signs of mild cognitive impairment or mild Alzheimer's disease but without amyloid deposits on positron emission tomography: Results from Bio‐Hermes Study participants

**DOI:** 10.1002/alz.71085

**Published:** 2026-01-28

**Authors:** Richard C. Mohs, Douglas W. Beauregard, Lynne Hughes, Cyndy B. Cordell, Allan I. Levey, Saima Rathore, Nicholas T. Seyfried, Erik C. B. Johnson, Jessie Nicodemus‐Johnson, Joshua Christensen, Robin Wolz, John Dwyer

**Affiliations:** ^1^ Global Alzheimer's Platform Foundation Washington District of Columbia USA; ^2^ Department of Neurology Emory University School of Medicine Atlanta Georgia USA; ^3^ Pentara Corporation Millcreek Utah USA; ^4^ IXICO PLC London UK

**Keywords:** AD, alzheimer's disease, alzheimer's disease novel blood‐based biomarkers, amyloid beta, bio‐hermes study, cytokines, NfL, non‐amyloid‐related cognitive impairment, proteomics, underrepresented populations

## Abstract

**INTRODUCTION:**

Alzheimer's disease (AD) study participants may present with cognitive impairment who do not have brain amyloid deposits (Aβ−). Identifying predictive biomarkers for non‐amyloid‐related CI may provide better screening tests for trials seeking only CI Aβ+ participants and new therapy targets.

**METHODS:**

Analysis of the Bio‐Hermes biomarker database identified subpopulations of clinically normal, CN Aβ− (*n = *313), CI Aβ− (*n* = 296), and CI Aβ+ (*n =* 258), and CN Aβ+ (*n = *84) participants. Comparative analysis of demographics, clinical assessments, biomarkers, cytokines, and proteomics results was conducted.

**RESULTS:**

Subgroup comparison of CI Aβ− versus CN Aβ− found that neurofilament light most clearly differentiated CI Aβ− from CN Aβ− participants. No other biomarker analysis reached a level of differential significance.

**DISCUSSION:**

Analyses showed many novel biomarkers do not differentiate CI Aβ− from CN Aβ−. New biomarkers are needed to best determine the neuropathology of the clinical presentation of AD.

**Highlights:**

- NfL differentiated CN Aβ− versus cognitively impaired Aβ−.
- Proteomics (two platforms) did not differentially assess cognitively impaired Aβ−‐.
- Many novel biomarkers did not differentially assess cognitively impaired Aβ−.
- New biomarkers are needed to determine the neuropathology of AD clinical presentation.

## 1 BACKGROUND

Participants evaluated for participation in clinical trials of new treatments for Alzheimer's disease (AD) are usually characterized by clinical evaluation and biomarkers. Trials may seek to enroll participants who are determined by clinical measures to be cognitively normal (CN), have mild cognitive impairment (MCI), or have dementia, possibly due to AD. The neuropathological status of study participants is usually characterized by biomarkers evaluating participants within the A (amyloid), T (tau), N (neurodegeneration) framework.^1^ The most well‐validated biomarkers for these neuropathological features are brain imaging measures, particularly amyloid^2^ and tau positron emission tomography (PET) scans,^3^, ^4^ and structural magnetic resonance imaging (MRI) scans.^5^ Recent advances in the development of blood‐based biomarkers have identified several that are also good measures of neuropathology, specifically, plasma phosphorylated tau (p‐tau) 217, which is highly predictive of both brain amyloid^6^, ^7^ and abnormal tau.^7^, ^8^ Neurofilament light (NfL) and total tau (t‐tau) have also been proposed as measures of neurodegeneration,^9^, ^10^ glial fibrillary acidic protein (GFAP) reflecting reactive astroglyosis,^11^, ^12^ and various cytokines^13^ have^13^ been proposed as measures of brain inflammation. Other blood biomarkers that may be associated with neurodegenerative disease are also being studied, including those associated with vascular damage^14^ and alpha‐synuclein, a protein associated with Lewy bodies in Parkinson's disease and Lewy body dementia.^15^ Coupled with clinical measures, the use of appropriate biomarkers enables study sponsors to enroll participants who have brain amyloid deposits, tau deposits, and clinical signs that are characteristic of patients at a particular stage in the progression of AD.

During the screening process for AD clinical trial enrollment, in most cases, participants are prescreened and excluded if there is clinical or laboratory evidence of brain disease other than AD, such as those with clear evidence of stroke, Parkinson's disease, alcohol or substance abuse, unstable medical illness, or active psychiatric disease. For prospective participants who proceed with clinical screenings, many who are identified as having clinical signs and symptoms consistent with the early stages of AD do not have biomarker results consistent with the neuropathologic features of AD and, thus, are not trial eligible. Recent trials seeking to enroll patients with early AD found screen failure rates of over 50%, with many of the screen failures resulting from clinically screened persons who do not have brain amyloid deposits as assessed by a brain PET scan.^16^, ^17^, ^18^, ^19^

The neuropathological substrate underlying the clinical signs of cognitive impairment (CI) in these screen failures is usually not clear. Autopsy studies looking at the brains of patients who die with a current or past clinical diagnosis of AD or MCI indicate that less than half of such patients have uncomplicated AD neuropathological change (ADNC), that is, amyloid plaques and tau tangles without other neuropathology.^20^ Commonly observed co‐pathologies are vascular changes, including atherosclerosis, large and small infarcts, neocortical Lewy bodies, age‐related tau deposits, and other neurodegenerative changes such as hippocampal sclerosis.^21^ In most instances, these other neuropathologies exist in combination with some level of amyloid plaques and neuronal tau, although some cases of suspected non‐amyloid pathology (SNAP) are observed.^20^

A previous paper^22^ presented biomarker results for the racially and ethnically diverse Bio‐Hermes study participants screened as they would be for enrollment in a typical AD clinical trial. The purpose of the present analyses is to evaluate blood‐based biomarkers among these study participants who, on clinical examination, met criteria for either MCI^23^ or mild AD^24^ but who were not amyloid positive as assessed by brain amyloid PET scan or, in a few cases where PET was not available, cerebrospinal fluid (CSF) measures.

## 2 METHODS

### 2.1 Participants and study flow

**RESEARCH IN CONTEXT**

1. **Systematic review**: Clinically, AD presents CI whereas amyloid deposits and tau tangles characterize the neuropathology. Some patients with CI do not have underlying AD neuropathology. We investigated blood‐based biomarkers potentially indicative of non‐amyloid‐related CI among the Bio‐Hermes community‐based, diverse population study participants.
2. **Interpretation**: Biomarkers compared included phosphorylated tau 217 and total tau, NfL, GFAP, and proteomics between subpopulations based on their cognition and amyloid status. Elevation of NfL was the biomarker that most clearly differentiated the participants who had Aβ− clinical CI from those who were CN and Aβ−. Furthermore, NfL was also elevated in the CI Aβ+ group relative to the CN Aβ+, indicating NfL is associated with CI along with Aβ+.
3. **Future directions**: This analysis highlights the need for additional biomarkers to identify neuropathologies underlying CI not associated with Aβ+.

The overall design of the Bio‐Hermes study, including recruitment strategy, visit structure, and inclusion and exclusion criteria, was described in a previous publication.^22^ Briefly, a total of 1001 participants were recruited across 17 clinical sites that regularly conduct therapeutic drug trials related to AD. These participants were selected from 1296 participants screened to meet clinical criteria for one of three groups. CN participants had no evidence of memory impairment on the delayed recall portion of the Rey Auditory Verbal Learning Test (RAVLT) test^25^ and no evidence of functional impairment on the Functional Assessment Questionnaire (FAQ).^26^ Persons with MCI met the clinical criteria described in Albert et al. ^23^ with memory impairment on the RAVLT (at least 1 standard deviation [SD] below age and race‐adjusted norms) but no functional impairment sufficient to interfere with Activities of Daily Living. Persons with mild AD met the clinical criteria described in McKhann et al.,^24^ having both memory impairment and a history of progressive functional impairment. Mild AD participants had a memory impairment on the RAVLT and a score on the MMSE of 20 to 24 and a small number of participants scoring less than 20 on the MMSE were admitted based on the investigators’ clinical judgement. Prospective participants with any active psychiatric or neurological condition other than AD were excluded, as were persons with a history of stroke, Parkinson's disease, or any unstable medical condition that might affect cognitive or functional assessment.

Participants who met the inclusion criteria for one of the three clinical groups were referred for a brain amyloid PET scan using the Amyvid (florbetapir) tracer. The scans were performed according to the product label.^27^ All images were uploaded for central reading by specialists trained in the reading of florbetapir images by IXICO; these experts had access to the standardized uptake value ratio (SUVR) but made a dichotomous classification as amyloid positive or negative according to the manufacturer's standards. One research site did not have access to a PET imaging facility and performed CSF studies for 11 participants; measurements of Aβ40, Aβ42, and the Aβ42/ Aβ40 ratio were determined by Quest Diagnostics, and the results were classified as being amyloid positive or negative.

Further details on the structure of study visits and the procedures for PET and CSF studies can be found in Mohs et al.^22^ The results of total tau and p‐tau217 and their relationship to participants’ amyloid PET/CSF status, as well as their clinical group membership and racial and ethnic differences, are also presented in Mohs et al.^22^

### 2.2 Collection and handling of blood samples

Blood samples for the measurement of biomarkers and for proteomics analyses were collected in K2 EDTA tubes and centrifuged at 1500 (± 100) × g at room temperature for at least 15 min. After centrifugation, plasma samples were placed in Simport tubes for transfer to various laboratories for analyses. P‐tau217 values were obtained using a Meso Scale Discovery immunoassay from the Clinical Diagnostics Laboratories of Eli Lilly and Co.^6^ A second p‐tau217 assay was performed by the University of Gothenburg using the commercial ALZpath p‐tau217 assay.^28^ Total tau (t‐tau), GFAP, and NfL assays were performed by Quanterix on an HD‐X Automated Immunoassay analyzer using commercially available Single Molecule Array (Simoa) Assay Kits in accordance with the recommendations from the manufacturer.^29^, ^30^ A second set of assays for NfL and GFAP was performed by Roche Diagnostics using the Elecsys immunoassay platform.^31^, ^32^ Cytokine analyses were performed by Merck Research Laboratories using FirePlex immunoassays^33^ generating a panel of 69 cytokine values. Merck also performed separate analyses for NfL and GFAP using the FirePlex immunoassays.^33^ The proteomics analyses, including SomaLogic SomaScan 7k proteomic platform^34^ and tandem mass tag‐mass spectrometry (TMT‐MS) platforms, were done at Emory University in collaboration with EMTherapro.^35^ TMT‐MS was analyzed on the top 14 depleted plasma samples without fractionation, as described in an earlier study.^36^

### 2.3 Quantitative analysis of amyloid PET images

Study participants were classified as amyloid positive or amyloid negative based on expert visual read of the amyloid PET scans as previously described.^22^ To investigate whether some of the CI Aβ− participants may have had subthreshold or regionally restricted amyloid deposits, the scans were also analyzed by brain region according to the method of Grothe et al.^37^

### 2.4 Analysis of biomarkers potentially indicative of non‐amyloid‐related CI

To investigate biomarkers potentially indicative of non‐amyloid‐related CI, we compared blood‐based biomarkers in cognitively impaired (MCI + Mild AD), amyloid‐negative participants (MCI + Mild AD Aβ−), with biomarkers in CN, amyloid negative participants (CN Aβ−), and with biomarkers in cognitively impaired amyloid‐positive participants (MCI + Mild AD Aβ+). We analyzed the amyloid PET and plasma p‐tau217 results to determine the extent to which there were subthreshold markers of amyloid and tau in the CI Aβ− group. We also analyzed blood markers of neurodegeneration and cytokine markers of inflammation. Lastly, because there could be other indicators of neuropathology underlying CI in the MCI + AD Aβ− participants, we analyzed the entire proteomics panel of SomaLogic and TMT‐MS proteins in an exploratory fashion to determine whether other abnormalities could be identified.

### 2.5 Statistical analysis

Analyses of each of the clinical and biological variables compared participants in the four study groups: CN Aβ−, MCI + Mild AD Aβ−, CN Aβ+, and MCI + Mild AD Aβ+. For demographic, clinical, and most biomarker variables, the initial comparison of groups was done by Pentara Corporation using SAS version 9.4 or R version 4.2.2 or higher. Comparisons accounted for sex, age, and years of education. In cases where the initial analysis showed significant group differences, follow‐up comparisons were done, adjusting for multiple comparisons.

The proteomics dataset generated from the SomaLogic SomaScan and TMT‐MS platforms was analyzed independently in an exploratory fashion. The R package SomaDataIO was used to read in and process the SomaLogic data. SomaScan data were analyzed using linear regression analyses performed with the lm function in base R. Volcano and density plots, as well as additional images, were programmed using custom code built in base R.  Principal component analysis identified the site as strongly associated with the proteomic profile. Site (fixed effect) was regressed out of the data using linear regression. The residuals were used for subsequent analyses. For SomaLogic SomaScan proteomics, comparisons between the CN Aβ− participants and other groups were performed using linear regression, adjusting for sex, race, age, years of education, and robust multiarray average (RMA). For TMT‐MS proteomics, batch effects were addressed using the in‐house TAMPOR (Total Abundance Median POrtioning and Re‐centering) normalization method,^38^ and group comparisons were similarly conducted using linear regression, adjusting for sex, race, age, and years of education. *P* values from the linear model were adjusted for multiple testing using the method of Benjamini and Hochberg.^39^ For group comparisons of interest and with significant group differences, the five most upregulated and five most downregulated proteins were identified.

## 3 RESULTS

### 3.1 Demographic and clinical

At the time of this analysis, participants deemed eligible for study inclusion identified 397 persons who were CN and 554 who met clinical criteria for cognitively impaired (MCI + Mild AD). There were 296 participants with either clinical MCI or mild AD who were not amyloid positive based on PET or CSF results. Table 1 presents the clinical characteristics of the four groups of interest. The race and ethnicity comparison found more members of underrepresented populations (URPs) in the MCI + Mild AD Aβ− group compared with the CN Aβ− group. Relative to the CN Aβ− group, those in the MCI + Mild AD Aβ− group had worse scores on the MMSE, RAVLT, and FAQ and slightly fewer years of education. These two groups did not differ in the proportion of apoE Ɛ4 carriers. Comparison of the MCI + Mild AD Aβ− group with the MCI + Mild AD Aβ+ group (i.e., clinical and biological AD), the former was younger, less likely to be composed of apoE Ɛ4 carriers, and was less impaired on the MMSE, RAVLT, and FAQ. As reported previously,^22^ the presence of amyloid deposits was also associated with older age, lower MMSE and RAVLT scores, and higher FAQ scores. ApolipoproteinE (apoE) Ɛ4 carriers were more common among those with amyloid deposits and among those with CI. Additional demographic characteristics of the Bio‐Hemes study population were previously published.^22^

**TABLE 1:** Demographic and clinical variables by clinical and amyloid PET status.

	Amyloid negative		Amyloid positive	
Demographics and clinical variables	Cognitively normal (CN Aβ−)	Cognitively impaired MCI + Mild AD (MCI + Mild AD Aβ−)	Cognitively impaired MCI + Mild AD (MCI + Mild AD Aβ+)	Cognitively normal (CN Aβ+)
Total (N)	(*N* = 313)	(*N* = 296)	(*N* = 258)	(*N* = 84)
Age, mean (SD)	69.7 (6.23)	72.0 (6.91)	74.6 (5.96)	72.9 (6.46)
Female/male, *n*/*n*	194/119	160/136	135/123	47/37
Education, years, mean (SD)	15.8 (2.48)	15.2 (2.75)	15.2 (3.13)	15.6 (2.42)
Non‐Hispanic White, *n*, %	251 (80.2)	204 (68.9)	198 (76.7)	72 (85.7)
Non‐Hispanic Black, *n*, %	30 (9.6)	43 (14.5)	17 (6.6)	10 (11.9)
Hispanic, *n*, %	27 (8.6)	43 (14.5)	38 (14.7)	≤5 (1.2)
Other URPs, *n*, %	≤5 (1.6)	6 (2.0)	≤5 (1.9)	≤5 (1.2)
MMSE, mean (SD)	28.4 (1.48)	26.2 (2.67)	24.4 (3.07)	28.5 (1.36)
RAVLT, mean (SD)	47.9 (13.25)	35.8 (13.01)	32.7 (11.25)	46.8 (13.63)
FAQ, mean (SD)	0.6 (1.38)	5.0 (6.20)	7.8 (6.24)	1.1 (1.86)
apoE ε4 carrier/non carrier, n/n	81/231	58/233	165/90	48/36

Abbreviations: AD, Alzheimer's disease; APOE, apolipoprotein; CN, cognitively normal; FAQ, Functional Activities Questionnaire; MCI, mild cognitive impairment; MMSE, Mini‐Mental State Exam; PET, positron emission tomography; RAVLT, Rey Auditory Verbal Learning Test; SD, standard deviation; URP, underrepresented population.

### 3.2 Biomarkers

Table 2 presents means and SDs for several of the most common biomarker variables for each of the clinical groups. Table 3 presents the results of the statistical tests comparing differences between groups for each of the biomarker variables in Table 2. All biomarkers except for t‐tau showed highly significant differences between groups. The comparison of most interest between the MCI + Mild AD Aβ− group relative to the CN Aβ− group found that only the NfL measures were significantly different. The MCI + Mild AD Aβ− participants had higher NfL levels than those in the CN Aβ− group on all platforms. Among amyloid PET‐positive participants, NfL values were also higher among those in the MCI + Mild AD Aβ+ participants than in the CN Aβ+ participants.

**TABLE 2:** Blood‐based and PET biomarkers by clinical and amyloid status.

		Amyloid negative		Amyloid positive	
Blood Measure	Data Source	Cognitively normal CN Aβ− (*N* = 313)	Cognitively impaired MCI + Mild AD Aβ− (*N* = 296)	Cognitively impaired MCI + Mild AD Aβ+ (*N* = 258)	Cognitively normal CN Aβ+ (*N* = 84)
Biomarker	Lab	Mean (SD)	Mean (SD)	Mean (SD)	Mean (SD)
SUVR	IXICO	1.00 (0.09)	1.01 (0.10)	1.45 (0.23)	1.35 (0.20)
P‐tau217 (U/mL)	Lilly	0.17 (0.06)	0.21 (0.14)	0.44 (0.26)	0.28 (0.10)
P‐tau217 (pg/mL)	Gothenburg	2.11 (1.44)	2.49 (2.24)	4.81 (2.50)	3.50 (1.52)
T‐tau (ng/L)	Quanterix	2.19 (2.19)	2.71 (6.77)	2.04 (1.00)	2.17 (0.98)
NfL	Merck	23.59 (13.03)	33.77 (30.89)	40.45 (28.38)	28.97 (14.71)
	Quanterix	21.22 (12.17)	30.44 (31.57)	36.23 (27.03)	25.80 (13.04)
	Roche	2.76 (1.48)	3.57 (3.00)	4.59 (3.55)	3.24 (1.85)
GFAP	Merck	136.92 (68.83)	161.58 (157.38)	252.70 (118.24)	203.53 (102.95)
	Quanterix	122.23 (63.21)	146.53 (151.54)	230.16 (111.30)	181.38 (97.49)
	Roche	87.27 (44.26)	100.93 (95.06)	157.70 (66.83)	130.24 (72.99)

Abbreviations: AD, Alzheimer's disease; CN, cognitively normal; GFAP, glial fibrillary acetic protein; MCI, mild cognitive impairment; NfL, neurofilament light; PET, positron emission tomography; p‐tau217, phosphorylated tau 217, T‐tau, total tau; SD, standard deviation; SUVR, standardized uptake value ratio.

**TABLE 3:** Blood‐based biomarker comparison–adjusted *p* values for multiple comparisons.

Biomarker	Lab	CN Aβ+ versus CN Aβ−	MCI + Mild AD Aβ− versus CN Aβ−	MCI + Mild AD Aβ− versus MCI + Mild AD Aβ+	MCI + Mild AD Aβ− versus CN Aβ+	MCI + Mild AD Aβ+ versus CN Aβ−	MCI + Mild AD Aβ+ versus CN Aβ+
SUVR (*F* = 514.271; *p* < 0.0001)	IXICO	<0.0001	0.7483	<0.0001	<0.0001	<0.0001	<0.0001
P‐tau217 (*F* = 100.105; *p* < 0.0001)	Lilly	<0.0001	0.1702	<0.0001	0.0192	<0.0001	<0.0001
P‐tau217 (*F* = 79.512; *p* < 0.0001)	Gothenburg	<0.0001	0.3421	<0.0001	0.0004	<0.0001	<0.0001
T‐tau *F* = 1.809 (NS)	Quanterix	—	—	—	—	—	—
NfL (*F* = 13.051; *p* < 0.0001)	Merck	0.8066	0.0003	0.1354	0.2619	<0.0001	0.0059
NfL (*F* = 10.638; *p* < 0.0001)	Quanterix	0.9113	0.0011	0.2579	0.2539	<0.0001	0.0118
NfL (*F* = 12.873; *p* < 0.0001)	Roche	0.9197	0.0259	0.0029	0.5996	<0.0001	0.0018
GFAP (*F* = 34.157; *p* < 0.0001)	Merck	0.0010	0.3410	<0.0001	0.0415	<0.0001	0.0198
GFAP (*F* = 31.872; *p* < 0.0001)	Quanterix	0.0036	0.2866	<0.0001	0.1141	<0.0001	0.0106
GFAP (*F* = 36.774; *p* < 0.0001)	Roche	0.0002	0.4109	<0.0001	0.0093	<0.0001	0.0361

*Note*: All other cytokines compared for populations listed were not statistically significant.

Abbreviations: AD, Alzheimer's disease; CN, cognitively normal; GFAP, glial fibrillary acidic protein; MCI, mild cognitive impairment; Nfl, neurofilament light; NS, not statistically significant; P‐tau217, phosphorylated tau 217; T‐tau, total tau; SUVR, standardized uptake value ratio.

GFAP differed significantly among the clinical groups for all three of the assays, but the pattern of group comparisons was different from that for NfL. The MCI + Mild AD Aβ− participants did not differ from the CN Aβ− participants for any of the three GFAP assays. Follow‐up comparison showed only small differences in GFAP between participants who were MCI + Mild AD Aβ− compared with those who were CN Aβ+. Levels were slightly higher in the CN Aβ+ group; the direction of results was the same for all GFAP assays, but the group difference was most significant for the Roche assay (Table 3). For participants who were cognitively impaired (MCI + Mild AD) and for participants who were CN, GFAP levels (Table 2) were significantly higher in those who were Aβ+ (*p *< 0.0001 for all assays, Table 3). GFAP levels were higher among participants with CI and amyloid deposits (MCI + AD Aβ+) compared with those who were CN and had amyloid deposits (CN Aβ+).

Quantitative measures of amyloid load on PET scan (SUVR) were not different in the MCI + Mild AD Aβ− participants relative to those in the CN Aβ− group; as expected, all of the Aβ+ groups had higher SUVR values compared to all of the Aβ− groups (Table 3). Participants with CI and a positive visual PET read (MCI + Mild AD Aβ+) had higher SUVR values than participants in the CN Aβ+ group (Table 2). Regional analyses (not shown) using the method of Grothe et al.^37^ found that none of the individual regions derived from the Braak staging method^40^ showed significant differences between the CN Aβ− group versus the MCI + Mild AD Aβ− group; consistent with the visual reads, all regions showed higher amyloid values in those who were Aβ+ compared with those who were[Fig alz71085-fig-0001] Aβ−.

Results of the p‐tau217 tests showed differences consistent with those for the SUVR measures; specifically, the MCI + Mild AD Aβ− participants did not differ from the CN Aβ− participants, while p‐tau217 values were elevated in the amyloid PET‐positive groups when compared with the amyloid PET‐negative groups, and p‐tau217 was higher in the MCI + Mild AD Aβ+ participants than in the CN Aβ+ participants (Table 2). The group differences for the p‐tau217 values were very similar for the p‐tau217 data from Lilly Diagnostic Laboratories and from the University of Gothenburg.

### 3.3 Cytokines

Of the 69 cytokines examined in the Fireplex panel, only four showed an overall significant difference between the four groups; they were Interleukin‐2 receptor (IL‐2R) (*F* = 4.011, *p* = 0.0075); hepatic growth factor (HGF) (*F* = 3.253, *p* = 0.0212); macrophage migration inhibitory factor (MIF) (*F* = 4.156, *p* = 0.0062); and inflammatory chemokine (C‐C‐L8) (*F* = 3.202, *p* = 0.0227). Follow‐up comparisons adjusting for multiple comparisons found that only MIF was significantly different between the MCI + Mild AD Aβ− (12.55 ng/L, SD = 10.81) and the CN Aβ− participants (9.90 ng/L, SD = 5.17). None of the other pairwise comparisons between those two groups were statistically significant.

### 3.4 Proteomics

**FIGURE 1 alz71085-fig-0001:**
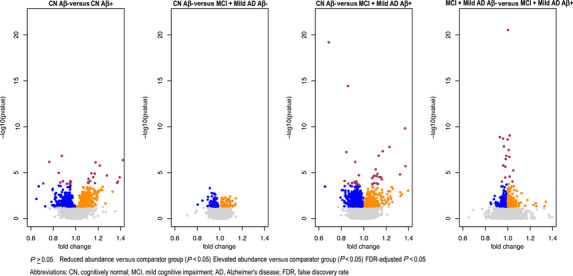
Volcano plot of protein abundance differences between groups defined by cognitive status (cognitively normal or mild cognitive impairment + mild Alzheimer's disease) and by amyloid positron emission tomography status (Aβ− or Aβ+). Dots represent individual proteins from SomaLogic SomaScan Platform.

Figure 1 presents the results of the discovery analysis of the entire set of proteomics data obtained from the SomaLogic SomaScan platform (TMT‐MS platform data only presented in Table 4). The Benjamini–Hochberg correction for multiple testing and a false discovery rate of < 0.05 revealed that there were no proteomics variables in data from both the platforms that significantly differentiated the participants in the CN Aβ− group from those in the MCI+AD Aβ− group. Group differences defined by amyloid PET status were associated with several proteins that were found in either increased or decreased abundance. Those most increased and decreased in each of these comparisons are listed in Table 4. Other manuscripts, including data from the Bio‐Hermes cohort, investigate the relationship of proteomics data to the presence of amyloid^41^ and to different APOE genotypes.^42^

**TABLE 4:** Ten proteins associated with the five most positive and five most negative differences.

SomaLogic proteins				Single‐shot TMT‐MS proteins			
Protein name	Description	*t* value	*p value*	Protein name	Description	*t* value	*p value*
**CN Aβ− versus CN Aβ+**				**CN Aβ− versus CN Aβ+**			
Most positive							
LRRN1‐CD	Leucine‐rich repeat neuronal protein 1:1‐13 Cytoplasmic domain	5.359	1.486E‐07	LDHB	Lactate dehydrogenase B	2.644	0.344
SPC25	Kinetochore protein Spc25	5.055	6.821E‐07	LDHA	Lactate dehydrogenase A	2.472	0.392
ALXO5	5‐Lipoxygenase	4.467	1.061E‐05	ASGR2	Asialoglycoprotein receptor 2	2.416	0.436
ATIII	Antithrombin‐III	3.978	8.384E‐05	APOF	Apolipoprotein F	2.381	0.456
FSD1	Acetylglucosaminyltransferase 1	3.969	8.706E‐05	PPIA	Peptidylprolyl isomerase A	2.264	0.522
Most negative							
BN3D2	Pre‐miRNA 5′monophosphate methyltransferase	−5.147	4.338E‐07	PAM	Peptidyl‐glycine alpha‐amidating monooxygenase	−3.552	0.240
NfL	Neurofilament light polypeptide	−5.028	7.783E‐07	CD163	Cluster of differentiation 163	−3.258	0.240
TBCA	Tubulin‐specific chaperone A	−4.864	1.712E‐06	ACE	Angiotensin‐converting enzyme	−3.249	0.240
SI8A	Alpha‐N‐acetylneuraminide alpha‐2, 8‐sialytransferase	−4.443	1.176E‐05	ALDOB	Aldolase, Fructose‐Bisphosphate B	−2.930	0.344
DCNL5	DCN1‐like protein 5	−4.423	1.285E‐05	APOE	Apolipoprotein E	−2.790	0.344
**CN Aβ− versus MCI + Mild AD Aβ+**				**CN Aβ− versus MCI + Mild AD Aβ+**			
Most positive							
SPC25	Kinetochoreprotein Spc25	9.515	6.361E‐20	ApoE4	Apolipoprotein E ε4	5.441	2.252E‐05
LRPN1‐CD	Leucine‐rich repeat neuronal protein 1: Cytoplasmic domain	8.105	3.658E‐15	APOD	Apolipoprotein D	3.957	0.007
CPLX2	Complexin‐2	5.504	5.782E‐08	AZGP1	Alpha‐2‐glycoprotein 1, zinc‐binding	3.478	0.025
CTF1	Cardiotrophin‐1	5.024	6.934E‐07	FGA	Fibrinogen α chain	3.458	0.025
FSD1	Fibronectin type III and SPRY domain‐containing protein 1	4.313	1.923E‐05	SERPINA1	Serpin family A member 1	3.441	0.025
Most negative							
BN3D2	Pre‐miRNA 5′monophosphate methyltransferase	−6.534	1.506E‐10	VTN	Vitronectin	−5.540	2.252E‐05
S100A13	Protein S100‐A13	−5.742	1.580E‐08	C3	Complement component 3	−4.667	6.257E‐04
TBCA	Tubulin‐specific chaperone A	−5.541	4.749E‐08	PLAT	Plasminogen activator, tissue type	−4.620	6.257E‐04
NfL	Neurofilament light polypeptide	−5.332	1.437E‐07	CAPS2	Ca2+‐dependent activator protein for secretion 2	−4.588	6.257E‐04
P5/11	Tumor protein p53‐inducible protein 11	−4.393	1.349E‐05	CFH	Complement factor H	−3.967	0.007
**MCI + Mild AD Aβ− versus MCI + Mild AD Aβ+**				**MCI + Mild AD Aβ− versus MCI + Mild AD Aβ+**			
Most positive							
SPC25	Kinetochoreprotein Spc25	9.904	2.976E‐21	ApoE4	Apolipoprotein E ε4	5.748	8.648E‐06
CPLX2	Complexin‐2	6.114	1.948E‐09	SERPINA1	Serpin family A member 1	3.010	0.172
CTF1	Cardiotrophin‐1	5.173	3.331E‐07	FGA	Fibrinogen alpha chain	2.881	0.214
LRRN1‐CD	Leucine‐rich repeat neuronal protein 1: Cytoplasmic domain	4.850	1.650E‐06	APOD	Apolipoprotein D	2.878	0.214
CBLH1	Cerebelin‐1	3.940	9.284E‐05	PON1	Paraoxonase 1	2.571	0.394
Most negative							
BN3D2	Pre‐miRNA 5′monophosphate methyltransferase	−6.244	9.041E‐10	C3	Complement component 3	−3.805	0.039
TBCA	Tubulin‐specific chaperone A	−6.177	1.346E‐09	APOE	Apolipoprotein E	−3.677	0.039
SERA	D‐3‐phosphoglycerate dehydrogenase	−6.072	2.488E‐09	VTN	Vitronectin	−3.660	0.039
S100A13	Protein S100‐A13	−5.768	1.397E‐08	CAPS2	Calcyphosine 2	−3.399	0.082
FOX01A	Foxhead box protein Spc25	−5.602	3.480E‐08	PLAT	Plasminogen activator, tissue type	−3.198	0.138

*Note*: Results show protein comparisons indicating whether levels in the first‐named group (e.g., CN Aβ−) are positive or negative relative to the second‐named group (e.g., CN Aβ+). Proteins selected after Benjamini–Hochberg correction (false discovery rate of < 0.05); no proteins were significantly associated with differences between CN Aβ− versus MCI + Mild AD Aβ−.

Abbreviations: AD, Alzheimer's disease; CN, cognitively normal; MCI, mild cognitive impairment.

## 4 DISCUSSION

The Bio‐Hermes study used a recruitment and screening process like that employed by sponsors of clinical trials investigating disease‐modifying agents for the treatment of AD. Of the 1001 participants enrolled in the study, 296 had clinical signs and symptoms consisted with MCI or AD but were not amyloid positive on PET or CSF (MCI +Mild AD Aβ−). The biomarker that most clearly differentiated this group from those who were CN and amyloid negative (CN Aβ−) was elevated NfL. This biomarker has been associated with neurodegeneration in several diseases, including AD, amyotrophic lateral sclerosis, multiple sclerosis, traumatic brain injury, and cerebrovascular disease.^43^ NfL may also discriminate neurodegenerative disease from psychiatric conditions in which the evidence for neurodegeneration is less clear.^44^ In the current study, NfL was also higher in the MCI + Mild AD Aβ+ group relative to the CN Aβ+ group, indicating that elevated NfL is associated with CI, even with background elevation associated with amyloid deposition. The fact that there was no significant elevation in markers of either amyloid pathology (SUVR) or tau pathology (p‐tau217, t‐tau) appears to rule out subtle increases in either of these neuropathological features as a basis for CI in this group.

Evidence for increased neuroinflammation in the MCI + Mild AD Aβ− participants was very modest. GFAP, which may reflect neuroinflammation,^43^ was not elevated in the MCI +Mild AD Aβ− group relative to the CN Aβ− group, and these groups were not markedly different on cytokine measures in the Fireplex panel, with the exception of MIF, which was elevated in the MCI + Mild AD Aβ− group. MIF has pleiotropic immune functions, including effects on cardiovascular and cerebrovascular processes^45^; further investigation of these mechanisms appears warranted. As in other studies, GFAP was associated with amyloid pathology (CN Aβ+ group vs CN Aβ− group; *p* < 0.004) and thus may be a useful biomarker reflecting the effects of anti‐amyloid treatments.^46^

The proteomics analysis did not reveal any single protein that was significantly increased or decreased in the MCI + Mild AD Aβ− group relative to the CN Aβ− group. One possible explanation for this is the biological heterogeneity within the MCI + Mild AD Aβ− group. Unlike Aβ+ individuals, whose pathology is more clearly defined by amyloid accumulation, Aβ− individuals with a clinical diagnosis of AD or MCI may represent a mixture of alternative disease mechanisms, each contributing variably across individuals. As a result, no single protein shows a strong, consistent association with CI in this group. Instead, multiple biological pathways may each contribute modestly in a subset of individuals, diluting the ability to detect robust group‐level differences. Other analyses pooling the Bio‐Hermes data with data from other studies found evidence for pathways that are either positively or negatively associated with amyloid deposition and with CI; these more complicated analyses are currently under review.^41^ Furthermore, it is likely that apoE genotype has a marked influence on plasma proteomics.^42^

Demographically the MCI + Mild AD Aβ− group was older and slightly less educated and had a higher proportion of persons from URPs than the CN Aβ− group. These “screen failures” were less likely to be apoE ɛ4 carriers than those in the MCI + AD Aβ+ group, further confirming that their underlying biological characteristics are not typical AD. The high prevalence of both Hispanic and African American participants in this group is consistent with findings from clinical trials showing high screen failure rates among members of URPs.^19^, ^46^

The results of this study highlight the need for additional biomarkers to identify specific pathologies underlying CI not associated with amyloid plaques and tau tangles. The elevation in NfL among these screen failures indicates neurodegeneration but does not indicate a more specific pathology that might be targeted with a new drug. Possible candidates are α‐synuclein pathology, trans‐activation response DNA‐binding protein 43 pathology, vascular damage, or other, as yet unknown, causes. Readily accessible biomarkers will be needed to identify patients with specific types of pathology and to enable the testing of novel drug treatments.

ClinicalTrials.gov ID: NCT04733989, A Biomarker Database to Investigate Blood‐Based and Digital Biomarkers in Participants Screened for Alzheimer's Disease (Bio‐Hermes). Sponsor, GAP Innovations, PBC.

## CONFLICT OF INTEREST STATEMENT

The authors have no conflicts of interest to declare. All authors have reviewed the contents of this manuscript, and there is no financial interest to report. Author disclosures are available in the Supporting Information.

## CONSENT STATEMENT

Prior to the start of any study procedures, informed consent was obtained from all study participants.

## Supporting information



Supporting information

## References

[1] Jack CR , Bennett DA , Blennow K , et al. A/T/N: an unbiased descriptive classification scheme for Alzheimer disease biomarkers. Neurology. 2016;87(5):539‐547. doi:10.1212/WNL.0000000000002923 27371494 PMC4970664

[2] Wilkins CH , Windon CC , Dilworth‐Anderson P , et al. Racial and ethnic differences in amyloid PET positivity in individuals with mild cognitive impairment or dementia: a secondary analysis of the Imaging Dementia–Evidence for Amyloid Scanning (IDEAS) cohort study. JAMA Neurol. 2022;79(11):1139‐1147. doi:10.1001/jamaneurol.2022.3157 36190710 PMC9531087

[3] Fleisher AS , Pontecorvo MJ , Devous MD , et al. Positron emission tomography imaging with [ ^18^ F]flortaucipir and postmortem assessment of Alzheimer's disease neuropathologic changes. JAMA Neurol. 2020;77(7):829. doi:10.1001/jamaneurol.2020.0528 32338734 PMC7186920

[4] Pontecorvo MJ , Devous MD , Kennedy I , et al. A multicentre longitudinal study of flortaucipir (18F) in normal ageing, mild cognitive impairment and Alzheimer's disease dementia. Brain. 2019;142(6):1723‐1735. doi:10.1093/brain/awz090 31009046 PMC6536847

[5] Jack CR , Knopman DS , Jagust WJ , et al. Tracking pathophysiological processes in Alzheimer's disease: an updated hypothetical model of dynamic biomarkers. Lancet Neurol. 2013;12(2):207‐216. doi:10.1016/S1474-4422(12)70291-0 23332364 PMC3622225

[6] Palmqvist S , Janelidze S , Quiroz YT , et al. Discriminative accuracy of plasma phospho‐tau217 for Alzheimer's disease vs other neurodegenerative disorders. JAMA. 2020;324(8):772‐781. doi:10.1001/jama.2020.12134 32722745 PMC7388060

[7] Mielke MM , Frank RD , Dage JL , et al. Comparison of plasma phosphorylated tau species with amyloid and tau positron emission tomography, neurodegeneration, vascular pathology, and cognitive outcomes. JAMA Neurol. 2021;78(9):1108‐1117. doi:10.1001/jamaneurol.2021.2293 34309632 PMC8314178

[8] Janelidze S , Berron D , Smith R , et al. Associations of plasma phospho‐tau217 levels with tau positron emission tomography in early Alzheimer disease. JAMA Neurol. 2021;78(2):149‐156. doi:10.1001/jamaneurol.2020.4201 33165506 PMC7653537

[9] Giangrande C , Delatour V , Andreasson U , Blennow K , Gobom J , Zetterberg H . Harmonization and standardization of biofluid‐based biomarker measurements for AT(N) classification in Alzheimer's disease. Alz & Dem Diag Ass & Dis Mo. 2023;15(3):e12465. doi:10.1002/dad2.12465 PMC10432775 37600860

[10] Hampel H , Gao P , Cummings J , et al. The foundation and architecture of precision medicine in neurology and psychiatry. Trends in Neurosciences. 2023;46(3):176‐198. doi:10.1016/j.tins.2022.12.004 36642626 PMC10720395

[11] Benedet AL , Milà‐Alomà M , Vrillon A , et al. Differences between plasma and cerebrospinal fluid glial fibrillary acidic protein levels across the Alzheimer's disease continuum. JAMA Neurol. 2021;78(12):1471. doi:10.1001/jamaneurol.2021.3671 34661615 PMC8524356

[12] Chatterjee P , Pedrini S , Doecke JD , et al. Plasma Aβ42/40 ratio, p‐tau181, GFAP, and NfL across the Alzheimer's disease continuum: a cross‐sectional and longitudinal study in the AIBL cohort. Alzheimers Dement. 2023;19(4):1117‐1134. doi:10.1002/alz.12724 36574591

[13] Morgan AR , Touchard S , Leckey C , et al. Inflammatory biomarkers in Alzheimer's disease plasma. Alzheimers Dement. 2019;15(6):776‐787. doi:10.1016/j.jalz.2019.03.007 31047856 PMC6565806

[14] Hinman JD , Elahi F , Chong D , et al. Placental growth factor as a sensitive biomarker for vascular cognitive impairment. Alzheimers Dement. 2023;19(8):3519‐3527. doi:10.1002/alz.12974 36815663 PMC10440207

[15] Yan S , Jiang C , Janzen A , et al. Neuronally derived extracellular vesicle α‐Synuclein as a serum biomarker for individuals at risk of developing Parkinson's disease. JAMA Neurol. 2024;81(1):59. doi:10.1001/jamaneurol.2023.4398 38048087 PMC10696516

[16] Budd Haeberlein S , Aisen PS , Barkhof F , et al. Two randomized phase 3 studies of aducanumab in early Alzheimer's disease. J Prev Alz Dis. 2022;9(2):197‐210. doi:10.14283/jpad.2022.30 35542991

[17] Honig LS , Vellas B , Woodward M , et al. Trial of Solanezumab for mild dementia due to Alzheimer's disease. N Engl J Med. 2018;378(4):321‐330. doi:10.1056/NEJMoa1705971 29365294

[18] Sperling RA , Donohue MC , Raman R , et al. Association of factors with elevated amyloid burden in clinically normal older individuals. JAMA Neurol. 2020;77(6):735‐745. doi:10.1001/jamaneurol.2020.0387 32250387 PMC7136861

[19] Van Dyck CH , Swanson CJ , Aisen P , et al. Lecanemab in early Alzheimer's disease. N Engl J Med. 2023;388(1):9‐21. doi:10.1056/NEJMoa2212948 36449413

[20] Abner EL , Kryscio RJ , Schmitt FA , et al. Outcomes after diagnosis of mild cognitive impairment in a large autopsy series. Ann Neurol. 2017;81(4):549‐559. doi:10.1002/ana.24903 28224671 PMC5401633

[21] Toledo JB , Arnold SE , Raible K , et al. Contribution of cerebrovascular disease in autopsy confirmed neurodegenerative disease cases in the National Alzheimer's Coordinating Centre. Brain. 2013;136(9):2697‐2706. doi:10.1093/brain/awt188 23842566 PMC3858112

[22] Mohs RC , Beauregard D , Dwyer J , et al. The Bio‐Hermes Study: biomarker database developed to investigate blood‐based and digital biomarkers in community‐based, diverse populations clinically screened for Alzheimer's disease. Alzheimers Dement. 2024;20(4):2752‐2765. doi:10.1002/alz.13722 38415908 PMC11032569

[23] Albert MS , DeKosky ST , Dickson D , et al. The diagnosis of mild cognitive impairment due to Alzheimer's disease: recommendations from the National Institute on Aging‐Alzheimer's Association workgroups on diagnostic guidelines for Alzheimer's disease. Alzheimers Dement. 2011;7(3):270‐279. doi:10.1016/j.jalz.2011.03.008 21514249 PMC3312027

[24] McKhann GM , Knopman DS , Chertkow H , et al. The diagnosis of dementia due to Alzheimer's disease: recommendations from the National Institute on Aging‐Alzheimer's Association workgroups on diagnostic guidelines for Alzheimer's disease. Alzheimers Dement. 2011;7(3):263‐269. doi:10.1016/j.jalz.2011.03.005 21514250 PMC3312024

[25] Rosenberg SJ , Ryan JJ , Prifitera A . Rey auditory‐verbal learning test performance of patients with and without memory impairment. J Clin Psychol. 1984;40(3):785‐787. doi:10.1002/1097-4679(198405)40:3<785::AID-JCLP2270400325>3.0.CO;2-4 6746989

[26] Pfeffer RI , Kurosaki TT , Harrah CH , Chance JM , Filos S . Measurement of functional activities in older adults in the community. J Gerontol. 1982;37(3):323‐329. doi:10.1093/geronj/37.3.323 7069156

[27] Lilly . Amyvid® Full Prescribing Information. Published online 2022. Accessed May 24, 2023. https://pi.lilly.com/us/amyvid‐uspi.pdf

[28] Ashton NJ , Brum WS , Di Molfetta G , et al. Diagnostic accuracy of a plasma phosphorylated tau 217 immunoassay for Alzheimer's disease pathology. JAMA Neurol. 2024;81(3):255. doi:10.1001/jamaneurol.2023.5319 38252443 PMC10804282

[29] Mendes AJ , Ribaldi F , Lathuiliere A , et al. Comparison of plasma and neuroimaging biomarkers to predict cognitive decline in non‐demented memory clinic patients. Alz Res Ther. 2024;16(1):110. doi:10.1186/s13195-024-01478-9 PMC11097559 38755703

[30] Simoa® Technology. Accessed April 15, 2025. https://www.quanterix.com/simoa‐technology/

[31] Roche's Elecsys sFlt‐1/PlGF ratio for predicting preeclampsia risk receives FDA 510(k) clearance. Accessed April 15, 2025. https://diagnostics.roche.com/us/en/home.html

[32] Mattsson‐Carlgren N , Collij LE , Stomrud E , et al. Plasma biomarker strategy for selecting patients with Alzheimer's disease for antiamyloid immunotherapies. JAMA Neurol. 2024;81(1):69. doi:10.1001/jamaneurol.2023.4596 38048096 PMC10696515

[33] FirePlex®‐96 Immunoassays. Accessed April 15, 2025. https://docs.abcam.com/pdf/fireplex/FirePlex‐96‐Immunoassays‐tech‐note.pdf

[34] SomaScan technology, with its leading 11K protein measurements, is the only precise proteomic tool. Accessed April 15, 2025. https://somalogic.com/somascan‐unique‐scale/

[35] Rayaprolu S , Higginbotham L , Bagchi P , et al. Systems‐based proteomics to resolve the biology of Alzheimer's disease beyond amyloid and tau. Neuropsychopharmacol. 2021;46(1):98‐115. doi:10.1038/s41386-020-00840-3 PMC7689445 32898852

[36] Johnson ECB , Dammer EB , Duong DM , et al. Large‐scale proteomic analysis of Alzheimer's disease brain and cerebrospinal fluid reveals early changes in energy metabolism associated with microglia and astrocyte activation. Nat Med. 2020;26(5):769‐780. doi:10.1038/s41591-020-0815-6 32284590 PMC7405761

[37] Grothe MJ , Barthel H , Sepulcre J , et al. In vivo staging of regional amyloid deposition. Neurology. 2017;89(20):2031‐2038. doi:10.1212/WNL.0000000000004643 29046362 PMC5711511

[38] Dammer EB , Seyfried NT , Johnson ECB . Batch correction and harmonization of–Omics datasets with a tunable median polish of ratio. Front Syst Biol. 2023;3:1‐13. doi:10.3389/fsysb.2023.1092341 PMC10137904 37122388

[39] Benjamini Y , Hochberg Y . Controlling the False Discovery Rate: a practical and powerful approach to multiple testing. J RStatist Soc B. 1995;57(1):289‐300. 10.1111/j.2517-6161.1995.tb02031.x

[40] Braak H , Braak E . Neuropathological stageing of Alzheimer‐related changes. Acta Neuropathol. 1991;82(4):239‐259. doi:10.1007/BF00308809 1759558

[41] Afshar S , Dammer EB , Bian S , et al. Plasma proteomic associations with Alzheimer's disease endophenotypes. Nat Aging. 2025;5(10):2104‐2124. doi:10.1038/s43587-025-00965-4 40931114 PMC12532695

[42] Li F , Chen Y , Western D , et al. APOE‐stratified proteomic and metabolomic analysis reveals that Alzheimer's disease is a metabolomic disorder driven by mitochondrial damage. Nature Aging. 2025;5(10):2104‐2124. doi:10.1038/s43587-025-00965-4 40931114 PMC12532695

[43] Hampel H , Hu Y , Cummings J , et al. Blood‐based biomarkers for Alzheimer's disease: current state and future use in a transformed global healthcare landscape. Neuron. 2023;111(18):2781‐2799. doi:10.1016/j.neuron.2023.05.017 37295421 PMC10720399

[44] Al Shweiki MR , Steinacker P , Oeckl P , et al. Neurofilament light chain as a blood biomarker to differentiate psychiatric disorders from behavioural variant frontotemporal dementia. J Psychiatr Res. 2019;113:137‐140. doi:10.1016/j.jpsychires.2019.03.019 30953863

[45] Zernecke A , Bernhagen J , Weber C . Macrophage migration inhibitory factor in cardiovascular disease. Circulation. 2008;117(12):1594‐1602. doi:10.1161/CIRCULATIONAHA.107.729125 18362243

[46] Sims JR , Zimmer JA , Evans CD , et al. Donanemab in early symptomatic Alzheimer's disease: the TRAILBLAZER‐ALZ 2 Randomized Clinical Trial. JAMA. 2023;330(6):512. doi:10.1001/jama.2023.13239 37459141 PMC10352931

